# Two-way communication between cell cycle and metabolism in budding yeast: what do we know?

**DOI:** 10.3389/fmicb.2023.1187304

**Published:** 2023-06-15

**Authors:** Yanfei Zhang, Lucas van der Zee, Matteo Barberis

**Affiliations:** ^1^Molecular Systems Biology, School of Biosciences, Faculty of Health and Medical Sciences, University of Surrey, Guildford, Surrey, United Kingdom; ^2^Synthetic Systems Biology and Nuclear Organization, Swammerdam Institute for Life Sciences, University of Amsterdam, Amsterdam, Netherlands; ^3^Centre for Mathematical and Computational Biology, CMCB, University of Surrey, Guildford, Surrey, United Kingdom

**Keywords:** cell cycle, metabolism, cyclin-dependent kinase, cyclins, metabolic enzymes

## Abstract

Coordination of cell cycle and metabolism exists in all cells. The building of a new cell is a process that requires metabolic commitment to the provision of both Gibbs energy and building blocks for proteins, nucleic acids, and membranes. On the other hand, the cell cycle machinery will assess and regulate its metabolic environment before it makes decisions on when to enter the next cell cycle phase. Furthermore, more and more evidence demonstrate that the metabolism can be regulated by cell cycle progression, as different biosynthesis pathways are preferentially active in different cell cycle phases. Here, we review the available literature providing a critical overview on how cell cycle and metabolism may be coupled with one other, bidirectionally, in the budding yeast *Saccharomyces cerevisiae*.

## 1. Introduction

*Saccharomyces cerevisiae*, commonly known as budding yeast, is the most thoroughly investigated eukaryotic microorganism, which aids our understanding of the biology of other eukaryotic cells like human cells (Ostergaard et al., 2000). Its cell cycle is relatively clear now. Most literature distinguishes four phases in the cell cycle, i.e., the G1 (Gap 1) phase with increasing cell size, the S (synthesis) phase with synthesis of DNA, the G2 (Gap 2) phase with rapid cell growth and protein synthesis, and the M (mitotic) phase with the condensation of chromosomal DNA and segregation of the sister chromatids. Besides the cycling states with these four different cell cycle phases, similarly to human cells, yeast cells may enter a quiescent state (known as G0), in which cells have arrested growth and are quiescent.

How can *S. cerevisiae* decide which state it will enter, and how should its reluctance to do be recognized as either a culture condition or a nutrient condition problem? Under nutrient limitations, such as carbon starvation, a cell can exit the active cell cycle and enter G0 (De Bruin et al., 2004; Gray et al., 2004). After a favorable change in nutrient availability such as with an extracellular increase of glucose, the cell will exit G0 and enter G1 (Zhang et al., 2019). How the cell decides when to enter the next cell cycle phase, can be recognized as a metabolic problem: here the cell must assess and coordinate its growth and metabolic states (Cai and Tu, 2012). For example, in G1 and S phases, a cell needs to direct its metabolism to synthesize enough proteins and DNA, respectively, before progressing into the M phase to generate a new daughter cell. In contrast, in the M phase DNA synthesis is inhibited.

The first example of temporal regulation of metabolism directly by the cell cycle has been published by Tu et al. (2007). It was reported that the concentration of numerous metabolites, such as amino acids, carbohydrates, and nucleotides, exhibit robust oscillations in what has been called the yeast metabolic cycle (YMC). Here, we shall refer to it as the yeast metabolic cell cycle, to distinguish it from the cycles of the equally metabolic ‘glycolytic oscillations’ with usual frequencies of around 1/min (Wolf et al., 2000). It is reported that the glycolytic flux peaks in G1 and reduces through S/G2 (Monteiro et al., 2019). A detailed study by Campbell et al. (2020) showed that protein synthesis mainly occurs in G1, while most metabolic pathways are active after cells commit to division in S and G2/M. An analysis of metabolite abundance showed that cells modulate precursor abundance according to their role in the metabolism. For example, amino acid abundance peaks in G1, nucleotides increase in S, and lipids and central carbon metabolism products peak at the end of the cell cycle in G2/M (Campbell et al., 2020). However, the detailed mechanisms by which metabolite concentrations influence the cell cycle, if they do, remain poorly understood. Empirically, metabolic enzymes affect the cell cycle. For example, a mutation in the *CDC30* (Phosphoglucose isomerase) gene causes cell cycle arrest late in the nuclear division (M phase; Dickinson and Williams, 1987); a *CDC35* (Adenylate cyclase) mutation causes cell cycle arrest in G1 (Casperson et al., 1985); a *CDC60* (Leucyl-tRNA synthetase) mutation results in cell cycle arrest at the nutrient control point START due to a deficiency of charged leucyl-tRNA (Hohmann and Thevelein, 1992); a *CDC64* (Alanyl-tRNA synthetase) mutation causes G1 arrest (Wrobel et al., 1999); and a *CDC8* (Thymidylate kinase) mutation interferes with DNA replication (Sclafani and Fangman, 1984). Despite this evidence, the more precise mechanisms through which inactivation of these metabolic enzymes induces cell cycle arrest are still unknown.

Here, we will critically discuss how recent progresses in *S. cerevisiae*, alias budding yeast, research enhance our understanding of how the cell cycle coordinates bidirectionally with metabolism.

## 2. Metabolic regulation of the cell cycle machinery

The building of a new cell is a very complex task that relies on thousands of metabolic and biosynthetic reactions to produce thousands of new proteins, lipids, and nucleic acids (Herrgård et al., 2008). In order to produce these big molecules, the cell needs to avail of enough biosynthetic precursors like carbon, nitrogen, phosphate. Otherwise, the growth of the yeast cells will be impeded and, most likely, it will then enter the G0 state which will enhance persistence and survival.

In budding yeast there is only one protein kinase (Cdc28 or Cdk1) that controls the initiation and completion of all four phases of the yeast cell cycle. The phase that it induces is determined by the cyclin protein that is associated with. Cln1 and Cln2 thereby serve to activate the budding at the G1/S transition. In parallel, Clb5 and Clb6 activate DNA synthesis in the S phase. Clb3 and Clb4 activate the transition to the G2 phase. Clb1 and Clb2 dictate the transition to the M phase. Cln3 directs the Cdk1 into a G1-specific transcriptional activation to initiate the cell cycle (Johnson and Skotheim, 2013), at the end of the G1 phase.

Many researchers believe that the levels of metabolic building blocks control the cell cycle at START. This point is an irreversible commitment to a new division cycle at the end of G1 (Charvin et al., 2010; Johnson and Skotheim, 2013). At START, Cln3 is released from the endoplasmic reticulum and the resulting Cln3/Cdk1 complex phosphorylates the transcription inhibitor Whi5, which stimulates its dissociation from the transcription factor complexes SBF and MBF (De Bruin et al., 2004). This results in the consequent weak transcriptional activation of two downstream G1 cyclin genes, *CLN1* and *CLN2* (De Bruin et al., 2004). The newly formed Cln1 and Cln2 pair with Cdk1 and direct the latter to phosphorylate Whi5, thereby inducing dissociation of more Whi5 from the transcription factor complexes SBF and MBF. The positive feed-forward loop that is thereby formed switches on the expression of more than 200 genes, resulting in an irreversible commitment to the cell cycle. The activated genes include the S phase cyclins *CLB5* and *CLB6* that can initiate DNA synthesis (Johnson and Skotheim, 2013).

**Figure 1:**
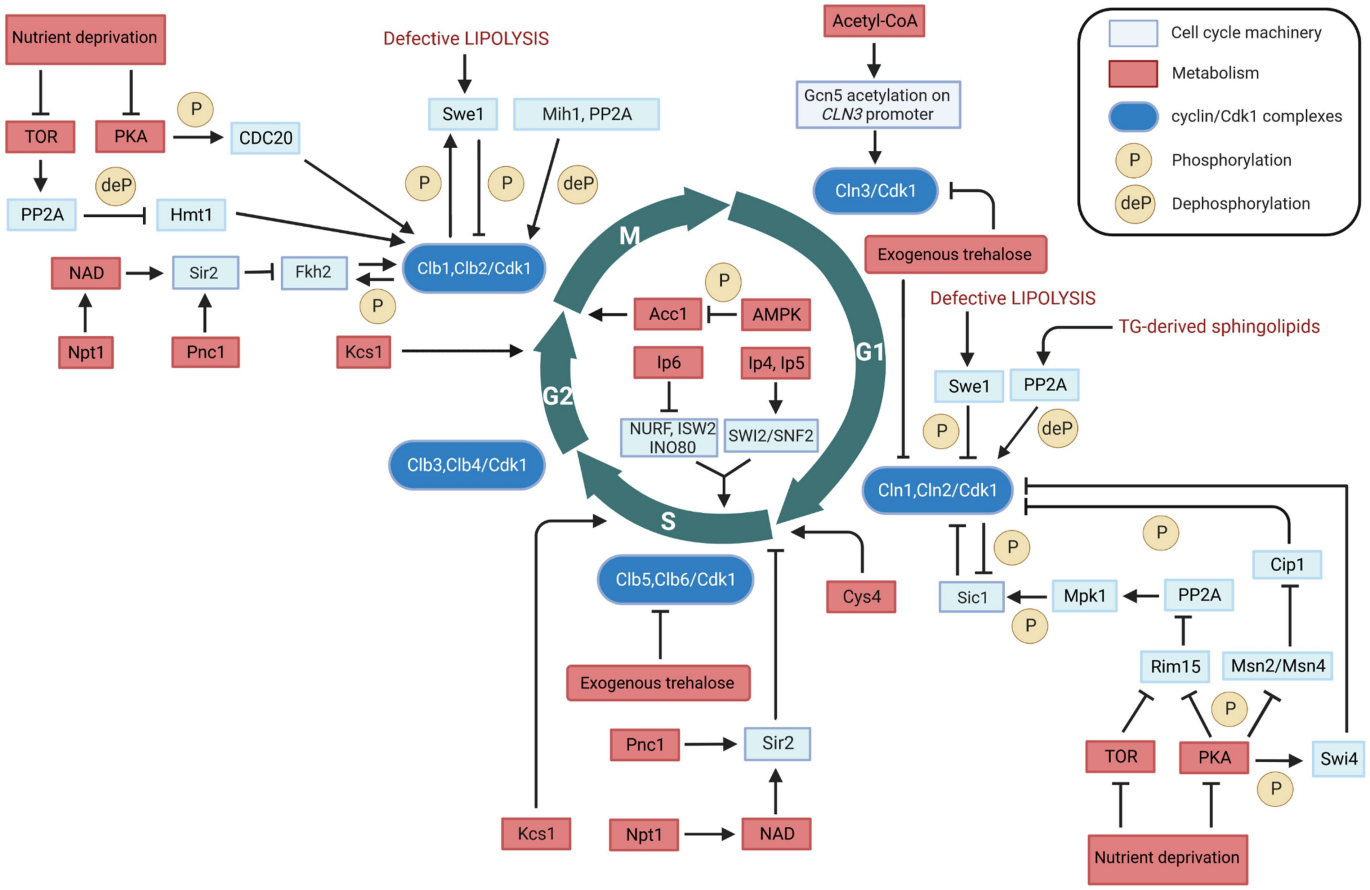
Metabolic regulation of cell cycle in *Saccharomyces cerevisiae*. Interactions between cell cycle players and metabolites/metabolic enzymes can be activatory (indicated by arrows) or inhibitory (indicated by blunt ended lines) and may be either direct or indirect. Color legend: light blue boxes indicate cell cycle regulators; red boxes indicate metabolic enzymes, pathways, or conditions; blue boxes indicate cyclin/Cdk1 kinase complexes; yellow circles with P represent phosphorylation events, whereas yellow circles with deP represent dephosphorylation events. Created with and adapted from BioRender.com.

Metabolic signaling through both the protein kinase A (PKA) pathway and the target of rapamycin (TOR) pathway constitutes the bridges between the nutrient status and START (Figure 1). Under carbon source starvation conditions, PKA and TOR are downregulated; this downregulation results in the upregulation of Rim15 (Moreno-torres et al., 2015). Rim15 inactivates the PP2A phosphatase activity, which normally dephosphorylates the phosphorylated Mpk1 mitogen activated kinase. The re-phosphorylated Mpk1 then phosphorylates and stabilizes Sic1 (Moreno-torres et al., 2015), which is the stoichiometric inhibitor of the mitotic Clb/Cdk1 complexes (Barberis et al., 2005). In turn, the stabilized Sic1 inhibits the activity of Clb5,6/Cdk1 (the cyclin-dependent kinase complexes that activate S phase), thus preventing S phase entry. Additionally, PKA phosphorylates the G1/S transcription factor Swi4, thereby inhibiting the transcription of *CLN1*, resulting in a transient G1 arrest (Amigoni et al., 2015). The inhibition of PKA and TOR pathways under nutrient-restrictive conditions also releases their suppression of Msn2/Msn4 activity. This increases the expression of the protein Cip1 and strengthens its inhibitory interaction with the cyclin Cdk1 complex (Chang et al., 2017).

Many researchers showed that TOR and PKA may also control M phase timing in yeast (Figure 1). Under nutrient-depictive condition, TOR activates PP2A which dephosphorylates the heterogeneous nuclear RNA-binding protein arginine methylthransferase Hmt1, causing a depletion of the B cyclin *CLB2* mRNA and a delay of M phase (Messier et al., 2013). PKA phosphorylates the mitotic inducer Cdc20, and the inactivation of PKA or expression of phosphorylation-defective *CDC20* promote Clb2 destruction (Searle et al., 2004).

Apart from the levels of metabolic ‘building blocks’, specific metabolic enzymes and/or their product metabolites can also regulate the cell cycle. In the following, we will describe these regulations in detail that are visualized in Figure 1.

### 2.1. Trehalose on G1 and S cyclins (indirect, G1 and S phases)

Trehalose is a non-reducing disaccharide composed of two glucose molecules that serves as a main carbohydrate storage in various organisms (Francois and Parrou, 2001). Apart from its role as a source of glucose, trehalose is involved in the cell cycle dynamics. At low sugar flux, trehalose levels increase during the G1 phase and decrease upon entry into the S phase (Schure et al., 1997); in addition, trehalose levels increase with decreasing growth rates (Silljé et al., 1999). Moreover, trehalose is a key determinant of the quiescent metabolic state that fuels cell cycle progression upon return to growth (Shi et al., 2010). Further evidence has been recently reported on the regulatory role of trehalose in the yeast cell cycle (Zhang et al., 2020). Specifically, administration of trehalose into the medium results in a significant inhibition of cell growth and increases the cell cycle doubling time (Table 1). The exogenous trehalose can decrease the intracellular concentration of glucose by inhibiting biosynthesis and degradation of trehalose (Zhang et al., 2020). The decreased intracellular concentration of glucose can cause the decline in glucose metabolism, thus leading to a decrease in Acetyl-CoA concentration which in turn can result in a decrease in the induction *CLN3* transcription (discussed in section 2.2). This may explain the fact that exogenous trehalose reduces the expression levels of the G1 cyclins genes *CLN1, CLN2* and *CLN3*, as well as of the S cyclins genes *CLB5* and *CLB* (Zhang et al., 2020). This evidence shows that exogenous trehalose may regulate the yeast cell cycle through induction of *CLN3* acetylation by regulating Acetyl-CoA concentration (Figure 1).

**Table 1:** Metabolic regulation of cell cycle in *Saccharomyces cerevisiae*.

Metabolite or enzyme	Cell cycle phase	Function of metabolite or enzyme	Cell cycle effect	Reference
Exogenous Trehalose	G1, S	Reduction of expression levels of G1 and S cyclin genes	Inhibition of cell growth and increase of cell cycle doubling time	Zhang et al. (2020)
Acetyl-CoA	G1	Induction of *CLN3* transcription by promoting acetylation of histones near its regulatory region	Signal for the initiation of cellular growth	Shi and Tu (2013)
Pnc1	G1, M	Conversion of nicotinamide (NAM) to nicotinic acid	Stimulation of Sir2 activity by preventing accumulation of NAM, by-product of Sir2	Gallo et al. (2004)
Npt1	G1, M	Catalyzation of NAD production	Regulation of Sir2 activity by maintaining a sufficiently high intracellular NDA concentration	Sandmeier et al. (2002)
Swe1	G1/S, M	Phosphorylation and inhibition of Cdk1 activity	Inhibition of cell cycling at multiple points	Chauhan et al. (2015)
Kcs1	S, G2/M	Catalyzation of inositol pyrophosphates production	Shortening the time from G1 to G2/M	Banfic et al. (2013)
Acc1	G2/M	Production of very long-fatty acids chain or derivatives required in the synthesis of membrane lipids	Localization to mitotic spindle poles and the cytokinesis furrow, and progression of cell division	Al-feel et al. (2003)
Cys4	G1, S	Catalyzing the production of cystathionine	Control of the G1 length and of the critical size at START	Hoose et al. (2012)

### 2.2. Acetyl-CoA on Cln3 (indirect, G1 phase)

Acetyl-CoA is a metabolite positioned at the intersection of many metabolic pathways. It arises in the transfer of an acetyl group from glucose, fatty acid, or certain amino acids to coenzyme A (CoA; Shi and Tu, 2015). In the glycolytic pathway for instance, glucose is broken down into two molecules of pyruvate. Then the pyruvate dehydrogenase complex catalyzes the oxidative decarboxylation of pyruvate to two molecules of acetyl-CoA, whilst reducing the redox coenzyme NAD^+^. Acetyl-CoA may then enter the TCA cycle, lipid synthesis or otherwise serve as C2 donor in anabolism. In addition to its function in cellular metabolism, acetyl-CoA is also a well-known substrate for acetylation reactions. In exponentially growing cells, acetyl-CoA levels are high but subsequently decrease upon entry into the stationary phase (Cai et al., 2011), suggesting that this metabolite is a critical metabolic signal for growth and proliferation. This change in acetyl-CoA levels may lead to alterations in the acetylation of proteins, which in turn modulate a variety of cellular processes, including gene expression, protein stability, and signaling pathways.

The effect of acetyl-CoA on growth and proliferation has been linked to the G1 cyclin Cln3, because acetyl-CoA induces *CLN3* transcription by promoting the acetylation of histones near its regulatory region (Shi and Tu, 2013) (Figure 1). The increased rate of intracellular acetyl-CoA synthesis upon initiation of growth (Cai et al., 2011) induces the acetylation of particular subunits within SAGA (an Spt-Ada-Gcn5-Acetyltransferase complex required for transcription in yeast) and enable the Gcn5 acetyltransferase to form a Gcn5p-containing Spt-Ada-Gcn5-acetyltransferase transcription coactivator complex. This coactivator complex catalyzes histone acetylation at the *CLN3* locus as well as around ribosomal and other growth genes (Table 1), thereby promoting cell cycling (Shi and Tu, 2013). Cln3 is an upstream activator of the G1 cyclins Cln1 and Cln2 (Tyers et al., 1993). The cyclin/Cdk1 complex in G1 phase is able to phosphorylate and activate the metabolic enzymes Gph1 and Nth1 (Ewald et al., 2016; Zhao et al., 2016), which convert the storage carbohydrate trehalose and glycogen into glucose, thus increasing the glucose metabolism. The increased glucose metabolism leads to an increase in the concentration of acetyl-CoA, which enhances transcription of *CLN3* and other growth genes, and accelerates cell growth.

Through this mechanism, acetyl-CoA links glycolysis to the cell cycle and may function as a signal for the initiation of cellular growth. The involvement of a regulator of cell cycle progression, i.e., Cln3, also impacts on the modulation of cellular metabolism (discussed in section 3).

### 2.3. Pnc1 and Npt1 on Sir2 (direct, G1 and M phases)

Sir2 is an NAD^+^-dependent histone deacetylase (HDAC) that functions in transcriptional silencing, and it has been associated with longevity (Kaeberlein et al., 1999). It appears in the middle of the M phase and disappears at the end of the G1 phase, and it has been shown to physically interact with the Forkhead transcription factors Fkh1 and Fkh2 that promote *CLB2* expression (Linke et al., 2013). Thus, Sir2 can regulate the level of the cyclin Clb2 through Fkh1/Fkh2 mediated binding to the *CLB2* promoter, with its deletion resulting in an increase of *CLB2* expression (Linke et al., 2013). Because Clb2 is necessary for Cdk1 activity in M phase, Sir2 contributes to the reduction of Clb2/Cdk1 activity at the end of M phase. However, Sir2 may also play a role at START; it can inhibit the assembly of the multiprotein complex necessary for the selection and activation of yeast replication origins, thereby inhibiting the initiation of DNA replication (Blank et al., 2008; Fox and Weinreich, 2008; Chang et al., 2011).

Nicotinamide (NAM) is a by-product of the Sir2 deacetylase reaction and can inhibit Sir2 activity. Pnc1 is a nicotinamidase that converts NAM to nicotinic acid; recombinant Pnc1 has been shown to stimulate Sir2 HDAC activity *in vitro* by preventing the accumulation of NAM produced by Sir2 (Gallo et al., 2004). The nicotinate phosphoribosyltransferase, Npt1, acts in the salvage pathway of NAD biosynthesis, and can regulate Sir2 activity by maintaining a sufficiently high intracellular NAD concentration (Sandmeier et al., 2002). Therefore, Pnc1 and Npt1 may regulate cell cycle progression by influencing the activity of Sir2 both at START and in M phase (Table 1; Figure 1).

### 2.4. Swe1 on Cdk1 (direct, G1/S and M phases)

Swe1, the yeast homolog of the mammalian kinase Wee1, links lipid metabolism with cell cycle *via* its effect on Cdk1 (Table 1). Swe1 phosphorylates Cdk1 and inhibits its kinase activity (Sia et al., 1996, 1998; Ubersax et al., 2003; Harvey et al., 2005). Swe1 can thereby potentially inhibit cell cycling at multiple points in the cell cycle, and its deletion leads to premature entry into mitosis that results in the birth of abnormally small cells (Harvey and Kellogg, 2003). Swe1 activity can be directly regulated by the Clb2/Cdk1 complex through an auto-regulatory loop between Swe1 and Clb2/Cdk1 (Harvey et al., 2005). Partial hyperphosphorylation of Swe1 stimulates its ability to phosphorylate Cdk1 which is required for the formation of the Swe1-Clb2/Cdk1 complex that inhibits Clb2/Cdk1. In this way, Swe1 binds and inhibits newly synthesized Clb2/Cdk1. This inhibition process is regulated by protein phosphatase 2A (PP2A), which sets a threshold that opposes the initial phosphorylation of Swe1 by Cdk1, thereby allowing a low constant level of Cdk1 activity to escape Swe1 inhibition in early mitosis (Harvey et al., 2011). Dephosphorylation of Cdk1 by the phosphatase Mih1 leads to further phosphorylation of Swe1 by Clb2/Cdk1 and to the release of Clb2/Cdk1 from the Swe1-Clb2/Cdk1 complex that can then rapidly trigger entry into mitosis (Harvey et al., 2005; Raspelli et al., 2018). Defective lipolysis activates Swe1, thereby halting cell cycle progression into M phase by phosphorylation of Cdk1 (Chauhan et al., 2015) (Figure 1). In addition to its function in modulating the phosphorylation state of Cdk1 to regulate the cell cycle in M phase, Swe1 together with Mih1 has been implicated in the control of mitotic spindle elongation (Raspelli et al., 2018).

The role of Swe1 has been reported also at the G1/S transition of the cell cycle, with Swe1 overexpression promoting a polarized cell growth (McCusker et al., 2007). Furthermore, Swe1 role may be linked to lipolysis (Chauhan et al., 2015). Swe1 is a lipid regulated kinase: deletion of Swe1 in a *tgl3 tgl4* lipase mutant restores cell cycle progression, whereas supplementation with saturated fatty acids or phytosphingosine, a precursor of sphingolipid synthesis, suppresses cell cycle delay in the lipase mutants (Chauhan et al., 2015). In cells with defective lipolysis, Swe1 delays the cell cycle at the G1/S transition by mediating the phosphorylation of Cln2/Cdk1 (Chauhan et al., 2015). Conversely, normal lipolysis (degradation) provides precursors for the synthesis of sphingolipids to activate the protein phosphatase PP2A (Fishbein et al., 1993; Nickels and Broach, 1996), which attenuates Swe1-mediated phosphorylation of Cdk1 by PP2A phosphatase activity on the phosphorylated Swe1 (Harvey et al., 2011). Besides its function in cell cycle progression, Swe1 also regulates sphingolipids biosynthesis (Chauhan et al., 2016), as well as Swe1 activity is regulated by sphingolipids through PP2A, thus an antiregulatory loop that controls cell cycle progression in response to alteration in sphingolipids metabolism. These mechanisms link lipolysis to cell cycle progression (Figure 1).

### 2.5. Inositol polyphosphates (indirect, S and G2/M phases)

Synthesis of inositol pyrophosphates through the activation of the hexakisphosphate and inositol heptakisphosphate kinase, Kcs1, plays an important role in the cell cycle progression. Kcs1 is responsible for an increase in the content of inositol pyrophosphates, which leads to progression through the S phase (Banfic et al., 2013). Overexpressed Kcs1 yeast cells arrested in G1 phase double the inositol pyrophosphates levels and reach the G2/M transition earlier than wild type cells (Banfic et al., 2016) (Table 1). Furthermore, inostitol pyrophosphates may regulate cell cycle by functioning in the process of vacuole biogenesis and cell wall integrity (Dubois et al., 2002), and regulating the Pho80-Pho85 cyclin/Cdk inhibitor complex Pho81, as yeast mutants defective in inositol heptakisphosphate (IP7) production are unable to inhibit the Pho80-Pho85 complex (Lee et al., 2007). Apart from the *in vivo* evidence of metabolic regulation of the cell cycle machinery, this regulation has also been shown by *in vitro* experiments. Eukaryotes use ATP-dependent chromatin-remodeling complexes to regulate DNA accessibility for transcription. There are four related classes of protein complexes (SWI2/SNF2, ISWI, Mi2 and INO80) which use the energy of ATP hydrolysis to alter nucleosome architecture. *In vitro* experiments show that inositol polyphosphates can modulate the activities of some chromatin-remodeling complexes. Specifically, inositol hexakisphosphate (IP6) inhibits nucleosome mobilization by the Nucelosome Remodeling Factor, NURF (ISWI ATPase-containing complex), the Imitation SWitch complex 2, ISW2 (ISWI ATPase-containing complex), and the INOsitol requiring, INO80, complexes (Shen et al., 2003). In contrast, nucleosome mobilization by the SWItch/Sucrose Non-Fermentable complex, SWI/SNF (SWI2/SNF2-containing complex) is stimulated by inositol tetrakisphosphate (IP4) and inositol pentakisphosphate (IP5; Shen et al., 2003). This evidence provides a link between inositol polyphosphate metabolism, chromatin remodeling, and gene expression (Figure 1).

### 2.6. Acc1 (direct, G2/M phase)

Acetyl-CoA carboxylase, Acc1, is a biotin-containing enzyme that connects lipid metabolism to the cell cycle. Acc1 catalyzes the carboxylation of acetyl-CoA to form malonyl-CoA, which plays an essential role in the synthesis and metabolism of fatty acids. Its connection with cell cycle is first reported 20 years ago (Al-feel et al., 2003). A temperature-sensitive *acc1*^ts^ mutant synchronized in G1 phase at the permissive temperature of 24°C and subsequently incubated at the restrictive temperature of 37°C, showed that 95% of the cell population became arrested at the G2/M transition despite the presence of fatty acids (C14-C26) in the medium (Al-feel et al., 2003). The cells also developed large undivided nuclei and the spindles of the arrested mutant cells were short (Al-feel et al., 2003). Supplementation of media with fatty acids of various chain lengths failed to support the growth of an *acc1* mutant strain, suggesting that this enzyme may function in producing long-chain fatty acids or derivatives thereof (Hasslacher et al., 1993). The translational efficiency of *ACC1* in mRNAs increases in G2/M, and this also reflects its protein levels (Blank et al., 2017). This evidence suggests that Acc1 plays an essential role in the progression of cell division and that external fatty acids cannot substitute for the products of the fatty acid synthesis pathway (Table 1), as the very long-chain fatty acids or derivatives may be required in the synthesis of membrane lipids. This mechanism links control of fatty acid synthesis to cell division (Figure 1).

Besides its metabolic-dependent regulation of the cell cycle, Acc1 also has a non-metabolic role. The cellular metabolic regulator AMP-activated protein kinase (AMPK) phosphorylates Acc1 on the serine 79 residue, inhibiting its enzymatic activity (Vazquez-martin et al., 2013). This inactive form of Acc1 has a role in the cell cycle, as it can localize to mitotic spindle poles and the cytokinesis furrow (Vazquez-martin et al., 2013).

### 2.7. Cys4 (indirect, G1/S phase)

Cystathionine-β-synthase, Cys4, catalyzes pyridoxal-phosphate-dependent (PLP) synthesis of cystathionine from serine and homocysteine (Banerjee et al., 2003), and it is one of the enzymes involved in sulfur metabolism. In the absence of Cys4, cells show cysteine auxotrophy (Thomas and Surdin-Kerjan, 1997) and cell proliferation can be delayed of about fourfold (Giaever et al., 2002). Reducing levels of Cys4 by disrupting the 3’ UTR of the *CYS4* gene compromises the ability of cells to undergo the metabolic cycle (Tu et al., 2007). Defects in this enzyme might not only hinder the production of antioxidants, such as glutathione, but also induce untimely and excessive production of S-adenosylmethionine, which could lead to aberrant methylation of macromolecules. A few studies have shown that Cys4 controls START: the introduction of a single hyperactive allele of Cys4 in wild type cells accelerates growth rate and initiation of DNA replication (START) (Blank et al., 2009), and loss of Cys4 delays START (Hoose et al., 2012) (Table 1). Besides shortening the G1 length of about 30%, Cys4 overexpression also reduced the critical size required at START (Hoose et al., 2012) (Figure 1).

## 3. Cell cycle regulation of metabolism

Under chemostat conditions, under which budding yeast becomes highly synchronized, more than half of the yeast genes exhibit periodic expression (Tu et al., 2005). Not only the genes show the oscillation but also the metabolites. NAD(P)H and ATP concentrations were found to oscillate with the cell cycle (Tu et al., 2005), as well as hundreds of metabolites change concentration during cell cycle progression (Ewald et al., 2016; Campbell et al., 2020). We recently explored possible novel regulations of the cell cycle engine on metabolic enzymes in budding yeast (Zhang and Barberis, unpublished). Fructose-1,6-bisphosphate aldolase (Fba1) and phosphoglycerate kinase (Pgk) are suspected to be phosphorylated by the Cyclin B/Cdk1, mammalian homologous of the budding yeast Clb2/Cdk1 (Zhang and Barberis, unpublished), suggesting that Fba1 and Pgk may also be phosphorylated by Clb2/Cdk1. Several papers published over the last few years showed other possible regulatory mechanisms, which are described below.

### 3.1. Cdk1 on Tgl4 (direct, late G1 phase)

**Figure 2:**
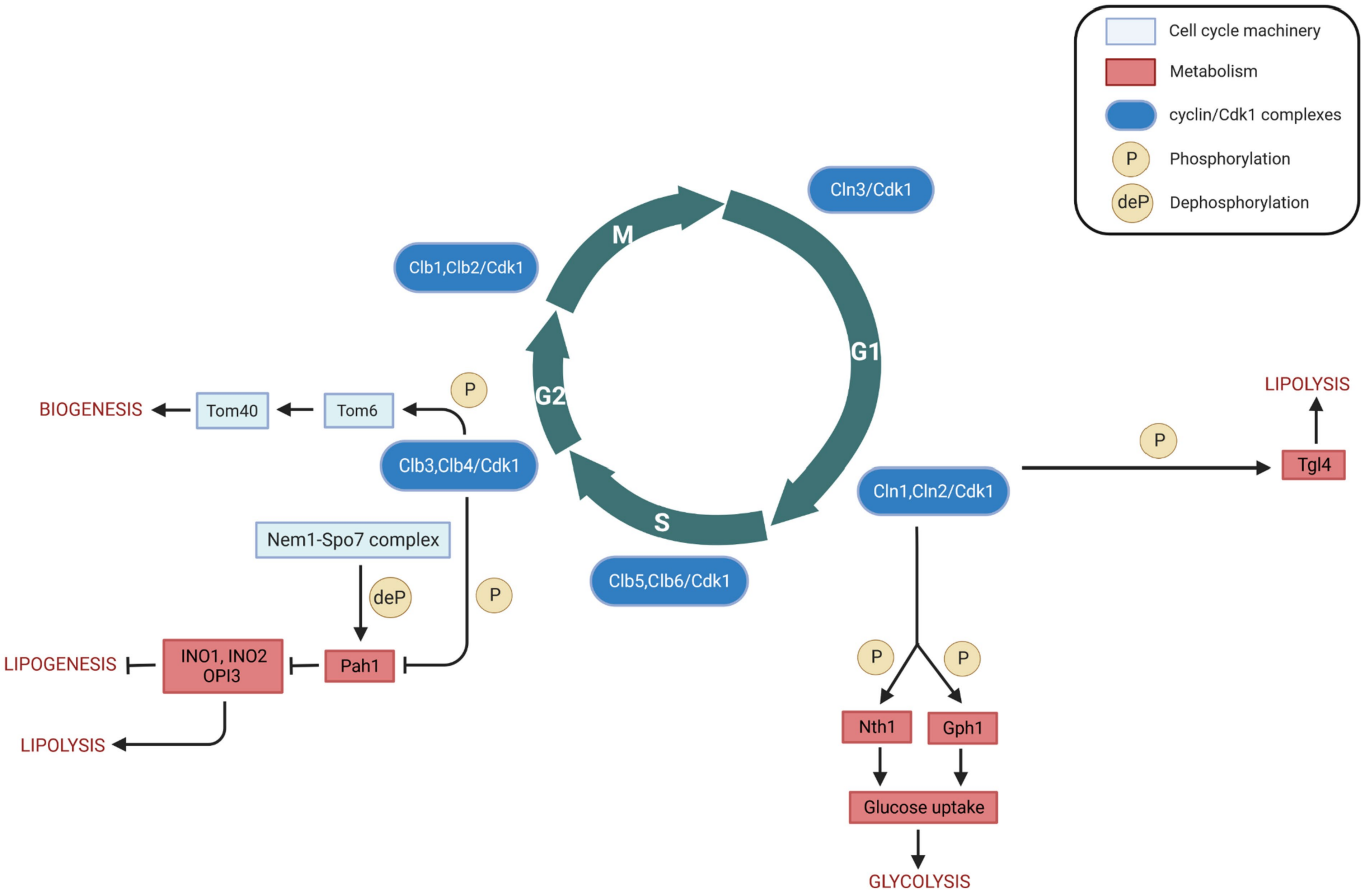
Cell cycle (cyclin/Cdk1) regulation of metabolism in *Saccharomyces cerevisiae*. Interactions between cell cycle players and metabolites/metabolic enzymes can be activatory (indicated by arrows) or inhibitory (indicated by blunt ended lines) and may be either direct or indirect. Color legend: light blue boxes indicate cell cycle regulators; red boxes indicate metabolic enzymes, pathways, or conditions; blue boxes indicate cyclin/Cdk1 kinase complexes; yellow circles with P represent phosphorylation events, whereas yellow circles with deP represent dephosphorylation events. Created with and adapted from BioRender.com.

Triacylglycerols (TGs) serve an essential cellular function as reservoirs for Gibbs energy substrates (fatty acids) and membrane lipid precursors (diacylglycerols and fatty acids). During cellular growth, the regulation of fatty acids and diacylglycerols is critical to maintain membrane lipid synthesis under energetically favorable conditions. Under stress conditions, yeast cells accumulate TGs which are stored in lipid droplets (Beopoulos et al., 2009). The first direct link between cell cycle and lipolysis showed that cells in the G0 phase exhibit a high lipid droplet content, and resumption of growth induces TG lipolysis (Kurat et al., 2006). A TG lipase could cleave the bound between the glycerol backbone and the fatty acids, which plays an essential role in balancing between stored TGs and free fatty acids. *S. cerevisiae* has mainly three TG lipases: Tgl3 (Athenstaedt et al., 1999), Tgl4 and Tgl5 (Athenstaedt and Daum, 2005). Among these, Tgl4 is the major functional ortholog of the murine adipose TG lipase ATGL (Kurat et al., 2009). It is phosphorylated and activated by Cdk1 (Ubersax et al., 2003), and the phosphorylation of Tgl4 is drastically reduced in mutants lacking the G1 cyclins Cln1 and Cln2, suggesting that Tgl4 phosphorylation is G1 cyclin-dependent (Kurat et al., 2009). The lack of TG degradation in *tgl3 tgl4* double mutants or the lack of Cdk1-mediated phosphorylation of Tgl4 can lead to delayed bud emergence and DNA synthesis (Kurat et al., 2009) (Table 2), suggesting that phosphorylation of Tgl4 can stimulate lipolysis, which contributes to early bud formation in late G1 phase. Moreover, Tgl4 is dephosphorylated upon entry into G1 phase (Nakatsukasa et al., 2022), indicating that its inactivation is also regulated in a cell cycle-dependent manner. Finally, cell cycle arrest induced at different phases leads to an increased lipid droplet content (Madeira et al., 2019). Taken together, these findings provide evidence for a direct metabolic link between lipolysis/TG degradation and cell cycle progression (Figure 2).

**Table 2:** Cell cycle (cyclin/Cdk1) regulation of metabolism in *Saccharomyces cerevisiae*.

cyclin/Cdk1 complex	Cell cycle phase	Metabolic target	Metabolic effect	Reference
Cln1,Cln2/Cdk1	G1	Tgl4	Stimulation of lipolysis which contributes to early bud formation in late G1 phase	Kurat et al. (2009)
Cln1,Cln2/Cdk1	G1/S	Nth1	Funneling the storage of carbohydrate trehalose into central carbon metabolism	Ewald et al. (2016)
Cln1/Cdk1	G1/S	Gph1	Funneling of the glycogen carbohydrate trehalose into central carbon metabolism	Zhao et al. (2016)
Clb3,Clb4/Cdk1	G2	Pah1	Driving of phospholipid biosynthesis required for nuclear membrane production	Santos-Rosa et al. (2005)
Clb3/Cdk1	G2/M	Tom6	Increase of the efficiency of Tom6 assembly into mitochondrial outer membrane, and stimulation of assembly of the protein import channel Tom40, which enhances the bioenergetics activity of mitochondria	Harbauer et al. (2014)

### 3.2. Cdk1 on Nth1 and Gph1 (direct, G1/S phase)

Trehalose and glycogen are the two glucose sources in yeast cells. Their possible role in cell cycle progression has been proposed by experiments showing that in synchronized cultures, under low sugar flux, trehalose and glycogen levels increase in G1 and are degraded upon entry into S phase (Schure et al., 1997). Furthermore, trehalose and glycogen accumulation depends on the duration of G1 phase; specifically, their accumulation decreases in *CLN3* overexpressing cells that have shorter G1 phase (Paalman et al., 2003). Trehalose and glycogen are also known to accumulate substantially in budding yeast upon entry into the G0 phase under unfavorable growth conditions (Boer et al., 2010; Shi et al., 2010). When in favorable growth conditions again, cells can use both the trehalose and the glycogen stores to help growth, but cells preferentially use trehalose (Shi et al., 2010). The regulation of these two carbohydrates by the cell cycle machinery is mediated by the Cdk1-mediated phosphorylation of the enzymes trehalase and glycogen phosphorylase (Table 2).

Two types of trehalase enzymes exist in budding yeast, i.e., the acid trehalase Ath1 (Alizadeh and Klionsky, 1996), and the neutral trehalase Nth1 (Kopp et al., 1993). Trehalose is mainly degraded by Nth1, which activation drives central carbon metabolism to the biosynthesis of proteins, lipids, and nucleic acids that are necessary for cell cycle progression (Ewald et al., 2016). Its deletion results in an abnormal cell cycle progression (Niu et al., 2008), indicating that Nth1 may be involved in the cell cycle. Clb2/Cdk1 obtained from yeast can phosphorylate Nth1 directly *in vitro*; at the G1/S transition, Cdk1 phosphorylates and directly activates Nth1 (Figure 2), which funnels the storage carbohydrate trehalose into central carbon metabolism (Ewald et al., 2016).

Gph1 (glycogen phosphorylase) is used in the breakdown of glycogen to glucose-1-phosphate (Francois and Parrou, 2001). Both Gph1 and Nth1 purified from *E. coli* can be phosphorylated *in vitro* by recombinant human Cyclin B/CDK1 (Zhao et al., 2016). Both glycogen and trehalose can accumulate in G1 phase when either G1 cyclin expression or Cdk1 kinase activity is blocked, and the glycogen and trehalose are degraded to glucose when Cln1/Cdk1 activity is provided (Zhao et al., 2016) (Figure 2). Therefore, Cdk1 can control the levels of glycogen and trehalose by phosphorylation of Gph1 and Nth1, thus allowing coordinated regulation of carbohydrate metabolism by cell division.

### 3.3. Cdk1 on Pah1 (direct, G2 phase)

Pah1 (previously known as Smp2), the budding yeast homolog of mammalian lipin, is a Mg^2+^ dependent phosphatidate (PA) phosphatase, which is a key enzyme of TG synthesis. It can catalyze the dephosphorylation of phosphatidic acid (PA) to yield diacylglycerol and inorganic phosphate (Han et al., 2006). Pah1 can be phosphorylated and inactivated by Clb3/Cdk1 and Clb4/Cdk1 complexes at the onset of mitosis (Ubersax et al., 2003; Santos-Rosa et al., 2005) (Table 2). As discussed above, Tgl4 is phosphorylated and activated by Cdk1 (Kurat et al., 2009). The opposite regulation of Pah1 and Tgl4 by Cdk1 results in the TG concentration to oscillate throughout the cell cycle: at the onset of bud formation and growth, phosphorylated Tgl4-catalyzed lipolysis becomes dominant to provide TG-derived precursors for membrane lipid synthesis, while Pah1 can synthesize TG for access *de novo* generated fatty acids. The phosphorylation of Pah1 may regulate Pah1 recruitment to the promoters of the lipid biosynthetic enzymes Ino1, Ino2, and Opi3, thereby driving phospholipid biosynthesis required for nuclear membrane production. This recruitment occurs in a Nem1-Spo7-dependent manner (Santos-Rosa et al., 2005; O’Hara et al., 2006). The Nem1-Spo7 complex serves as a phosphatase for Pah1, and Pah1 dephosphorylation can repress nuclear growth during interphase by blocking phospholipid biosynthesis (Santos-Rosa et al., 2005). Thus, Cdk1, the Nem1-Spo7 complex, and Pah1, together, may regulate phospholipid biosynthesis and nuclear membrane production that should then constitute another direct link between cell cycle and metabolism (Figure 2).

### 3.4. Cdk1 on Tom6 (direct, G2/M phase)

Mitochondria play a central role in cellular energy transduction, metabolism, and apoptosis. Most mitochondrial proteins are synthesized on cytosolic ribosomes and must be imported across the mitochondrial membranes (Chacinska et al., 2009). Up to 1,000 different proteins imported into mitochondria are essential for biogenesis of the organelle (Harbauer et al., 2014). A multi-subunit translocase of the outer mitochondrial membrane (TOM complex, the general entry gate) mediates both the import of mitochondrial precursor proteins into internal compartments of organelle and the insertion of proteins into the mitochondrial outer membrane (Rapaport, 2005; Dukanovic et al., 2009). The TOM complex is composed of at least seven different subunits. Tom 40, 22, 7, and 6 form the stable TOM core complex (Rapaport, 2005), with Tom 40 being the central component that forms the protein conducting channel of the complex (Charvin et al., 2010). Tom6 is a small integral membrane protein that plays a role in the assembly of the TOM complex (Dekker et al., 1998). The first link between Tom6 and cell cycle showed that Tom6 could be phosphorylated by recombinant mammalian kinases Cyclin B/CDK1 and Cyclin A/CDK2 (Schmidt et al., 2011). Furthermore, Tom6 mRNA level increases in G2/M phase, resulting in an increase of Tom6 in M phase (Harbauer et al., 2014). Phosphorylation of Tom6 is mediated by Clb3/Cdk1, and phosphorylated Tom6 stimulates its assembly into the mitochondrial outer membrane with an increased efficiency (Harbauer et al., 2014). Also, phosphorylated Tom6 stimulates the assembly of the protein import channel Tom40 (Harbauer et al., 2014) (Table 2). As the TOM complex is the entry gate for protein import into mitochondria, more and stable TOM complex can open the gates for the import of more proteins which could be used to build further mitochondria (Shiota et al., 2015). This process can lead to an increase in the energetic capacity of the oxidative phosphorylation machinery and to enhance the bioenergetics activity of mitochondria. Therefore, the phosphorylation of Tom6 in a cell cycle specific-manner and its involvement in the assembly of the TOM complex and protein import channel could have a significant impact on mitochondrial biogenesis (Figure 2).

Of note, during cell cycle progression, two regulatory mitochondrial fusion guanosine triphosphatases (GTPases), Fzol and Mgm1, were significantly increased, indicating a regulation of mitochondrial biogenesis (Harbauer et al., 2014). The increased import rates for Fzol and Mgm1 can lead to an increase in the energetic capacity of the oxidative phosphorylation machinery (Meeusen et al., 2006; Shiota et al., 2015), which enhances the bioenergetics activity of mitochondria.

## 4. Discussion

**Figure 3:**
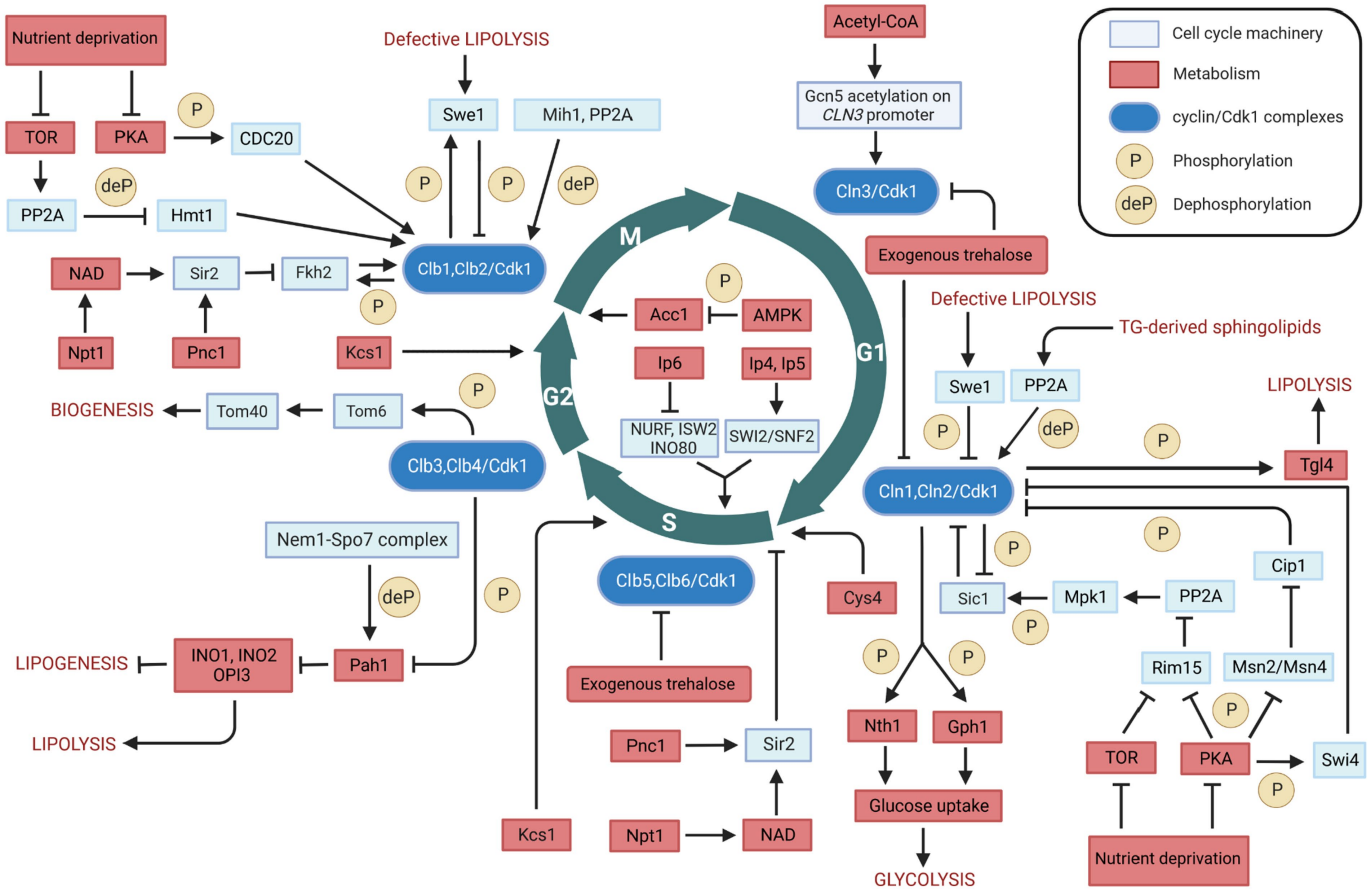
Overview of the bidirectional regulation between cell cycle and metabolism in *Saccharomyces cerevisiae*. Interactions between cell cycle players and metabolites/metabolic enzymes can be activatory (indicated by arrows) or inhibitory (indicated by blunt ended lines) and may be either direct or indirect. Color legend: light blue boxes indicate cell cycle regulators; red boxes indicate metabolic enzymes, pathways, or conditions; blue boxes indicate cyclin/Cdk1 kinase complexes; yellow circles with P represent phosphorylation events, whereas yellow circles with deP represent dephosphorylation events. Created with and adapted from BioRender.com.

Cdk1 is the most well-studied cell cycle protein. It has been shown to play an important role in coupling cell cycle with metabolic processes such as lipolysis, lipogenesis, glycolysis, and biogenesis. In this perspective, we discussed the available, limited evidence of the Cdk1-mediated biochemical processes at the interface between cell cycle and metabolism in the budding yeast *Saccharomyces cerevisiae* (Figure 3).

The regulation of metabolism and cell cycle is a complex, bidirectional process. The lipid metabolism is an example, being regulated in a bidirectional manner by Cdk1. On the one hand, Cdk1 phosphorylates and activates the Tgl4 at the onset of bud formation, resulting in the production of cell membrane precursors and energy substrates fatty acids from lipid triacylglycerol. Also, Cdk1 phosphorylates and inactivates Pah1 – a key enzyme for TG synthesis – in the G2 phase, resulting in an increased phospholipid synthesis that is required for nuclear membrane production. These observations indicate a cell cycle-dependent regulation of lipid metabolism, in which the same kinase controls both TG synthesis and degradation processes. On the other hand, lipid metabolism regulates the cell cycle. For instance, defective lipolysis can delay the G1/S transition and halt cell cycle progression into M phase by activating Cdk1-mediated phosphorylation of Swe1. In addition, the regulation of sphingolipids biosynthesis by Swe1 may represent an even more complex regulation between lipid metabolism and cell cycle. Furthermore, cells with defective Acc1 – a key enzyme that catalyzes the *de novo* synthesis of fatty acids – arrest at the G2/M transition. These observations highlight the bidirectional relationship between lipid metabolism and the cell cycle machinery.

The communication between carbohydrate metabolism and the cell cycle is also bidirectional. Cdk1 phosphorylates and activates Nth1 and Gph1, which convert the storage carbohydrates trehalose and glycogen into glucose. An increased glucose concentration, in turn, enhances the flux of glycolysis and the concentration of Acetyl-CoA. Acetyl-CoA plays a critical role in cell growth and cell cycle progression by enhancing *CLN3* transcription in G1 phase. Furthermore, the concentration of Acetyl-CoA can affect the cell cycle in G2/M phase, by influencing the *de novo* synthesis of fatty acids via Acc1. Moreover, Acetyl-CoA can also be transported and oxidized in mitochondria for ATP synthesis. The energetic capacity of oxidative phosphorylation in mitochondria can be further increased by the Cdk1-mediated phosphorylation of Tom6. These observations indicate that the relationship between carbohydrate metabolism and the cell cycle machinery is highly integrated and bidirectional.

Cell cycle progression is also being regulated by Cys4, which has been shown to play a crucial role in controlling the length of G1 phase. In this critical cell cycle phase, cells must synthesize a large number of proteins, and Cys4 catalyzes the synthesis of cystathionine, an essential precursor for the production of cysteine and glutathione. Cys4 deficiency can cause a shortage of cysteine and glutathione and induce the overproduction of SAM, resulting in the aberrant methylation of macromolecules such as newly synthesized histone, DNA, mRNA and lipids that are crucial for cell division. Furthermore, Cys4 delays cell cycle progression *via* its non-catalytic role, which results in the decrease of the critical cell size required at START. In this context, it may be worth exploring whether the effect of Cys4 on cell cycle progression may be mediated by Cdk1 phosphorylation.

Several other metabolic enzymes have been reported to play important roles in cell cycle regulation, through the proteins that can regulate Cdk1 activity or the expression of the cyclin subunits. For example, Pnc1 and Npt1 modulate the activity of Sir2, which regulates the expression level of Clb2 in M phase and inhibits DNA replication at START. Also, Kcs1 regulates the cell cycle *via* production of inositol pyrophosphates, which promote cell growth by modulating the activity of some chromatin-remodeling complexes, vacuole biogenesis, and cell wall integrity.

From the evidence summarized and discussed here, we conclude that various links between the cell cycle and the metabolism exist, in either direction. What is uncertain however, is whether all these links are always active or never active really. We speculate that most of the links are conditionally active, i.e., that their activity depends on the phase the cell cycle is in as well as on the state intermediary metabolism is in, and perhaps more directly on the extracellular nutrition conditions. As some of the mechanisms by which the cell cycle is coordinated with metabolism are likely to be conserved between unicellular and multicellular organisms, an overview of the communication mechanism of budding yeast can shed light on fundamental principles in mammalian cells, for example in controlling tumorigenesis. The complexity is then that in budding yeast as well as in mammalian cells the activities may be of various strengths, which will need to be assessed under the relevant conditions. The effects of the various activities then need to be integrated, where mathematical modeling may be an essential tool. This will provide substantial work for Systems Biology.

## Data availability statement

The original contributions presented in the study are included in the article. Further inquiries can be directed to the corresponding author.

## Author contributions

MB conceived and designed the study, provided scientific leadership, and supervised the study. YZ, LZ, and MB performed the literature search. YZ and MB wrote the paper. All authors contributed to the article and approved the submitted version.

## Funding

This work was supported by the Systems Biology Grant of the University of Surrey to MB, and by the SILS Starting Grant of the University of Amsterdam to MB. YZ was supported by a fellowship of the China Scholarship Council (CSC).

## Conflict of interest

The authors declare that the research was conducted in the absence of any commercial or financial relationships that could be construed as a potential conflict of interest.

## Publisher’s note

All claims expressed in this article are solely those of the authors and do not necessarily represent those of their affiliated organizations, or those of the publisher, the editors and the reviewers. Any product that may be evaluated in this article, or claim that may be made by its manufacturer, is not guaranteed or endorsed by the publisher.
